# Digital finance and migrant workers' urban integration: The mediation effect of the gender-earning gap

**DOI:** 10.3389/fpubh.2022.1076783

**Published:** 2022-12-19

**Authors:** Zhiheng Yang, Tiantian Liu, Yao Xiao

**Affiliations:** ^1^Institute of Regional Economics, Shandong University of Finance and Economics, Jinan, China; ^2^School of Economics, Shandong University of Finance and Economics, Jinan, China

**Keywords:** digital finance, migrant workers' urban integration, income, gender-earning gap, China

## Abstract

**Introduction:**

In the context of the wide application of digital finance, whether digital finance promotes or inhibits migrant workers' urban integration is an important issue.

**Methods:**

Based on microdata from the Chinese Social Survey (CSS) in 2019, we examined the mediation effects of inclusive digital finance on migrant workers' urban integration.

**Results:**

The empirical results showed that digital finance promotes migrant workers' integration into urban life and has positive effects. When the digital finance index increases by 1 unit, the urban integration of migrant workers also increases by 0.599 units. The usage depth and digitization degree of digital finance are positively correlated with the assimilation process of urban migrant workers, with coefficients of 0.690 and 1.282, respectively. Using the intermediary effect model, it was found that the development of digital finance promotes migrant workers' integration into urban society by narrowing the gender gap in income. One unit of digital finance increases the income of female migrant workers by 144.4% points greater than that of male migrant workers. It significantly improves the ability of female migrant workers to obtain wealth and promotes their integration into cities and family migration.

**Discussion:**

It is necessary to strengthen the promotion and utilization of digital finance to enhance its positive impact on the assimilation process of urban migrant workers by strengthening the construction of digital financial infrastructure, improving supporting policies related to the development of digital finance and improving the financial literacy of migrant workers, especially female migrant workers.

## 1. Introduction

The migration of populations from rural to urban areas, characterized by urbanization, is a great opportunity for developing countries. A large number of studies on labor migration showed that rural-urban migration contributes to social prosperity and progress. Relying on strong industrial and economic advantages, urbanization in China has achieved remarkable results. The urbanization rate of permanent residents was 64.72% in 2021, which was 33.35% greater than that at the end of 1978. Meanwhile, the urbanization rate of the registered residents in China was 46.7%. Two hundred sixty million migrant workers live in cities and towns, but their Hukou was still in the countryside. The transformation of rural migrants' working and living environments causes identity dislocation. Most of them struggle with the loss of rural identity and the formation of urban identity (1, 2).

Low income and employment instability are common problems in migrant workers' urban integration (3–6). Only migrant workers with strong bargaining power are likely to settle down when facing high housing prices and family migration costs (7, 8). It was found that the traditional definitions and methods of promoting migrant workers' urban integration are narrow because they only focus on some features of their human capital. The external social background that affects the integration of migrant workers, such as social inclusion resulting from technological progress, should also be considered and addressed (9–12).

In the era of rapid development of digital technologies such as the Internet, big data, cloud computing, and other information technologies, digital progress in the financial sector is making great strides (13–17). Since the launch of “Yu'e Bao” in 2013, digital finance in China has developed rapidly. According to the Peking University Digital Inclusive Finance Index (2011–2020) Report, the digital inclusive finance development index increased from 33.6 in 2011 to 334.8 in 2020, with an average annual growth of 29.1%. Digital finance can significantly reduce the cost of services provided by financial institutions and improve the penetration rate of financial services, thus greatly improving the availability of financial resources (18). A growing body of evidence to supports the claim that digital finance mainly spurs inclusive growth through wealth channels and innovation channels. For example, Durai et al. (19) argued that digital finance has the potential to provide affordable, convenient, and secure banking services that ensure access to financial services and adequate credit where needed by vulnerable groups. He and Li (20) confirmed that digital finance positively impacts farmers' entrepreneurial behavior, especially for groups with low human capital, material capital, and social capital. Li et al. (21) suggest that digital finance is positively correlated with food, clothing, house maintenance, medical care, education, and entertainment expenditures.

Did digital finance improve the economic conditions of rural-urban migrants and serve as a migration driver for migrant workers' urban integration? This is a question that remains to be studied. There is still a lack of empirical research on the impact of digital finance on migrant workers' urban integration, and there is no empirical research on how migrant workers can obtain urban integration dividends from digital finance.

Based on this question, we believe that an analysis of the impact of digital finance on migrant workers' urban integration by narrowing the gender-earning gap is necessary. The main contributions of the present study are as follows: we creatively connected digital finance with migrant workers' urban integration, took the gender-earning gap as a mediator to analyze whether there are gender disparities in the income distribution effect of digital finance, and analyzed this issue from coverage breadth, usage depth, and digitization degree, which enriches the research scope of the impact of digital finance on migrant workers' urban integration.

**Figure 1:**
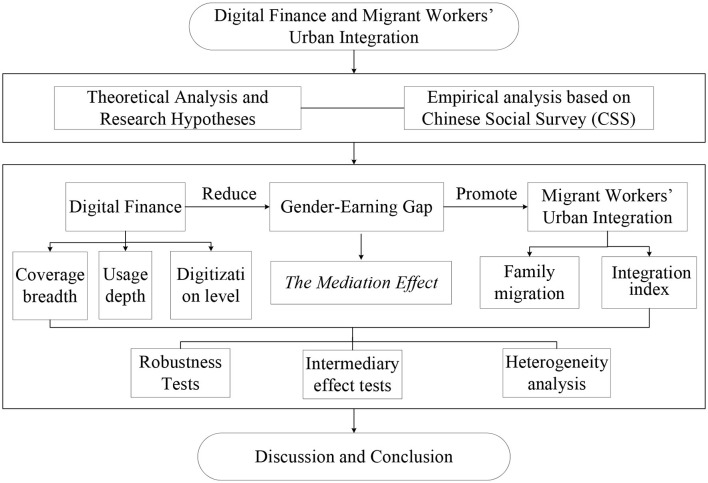
Technical roadmap.

The rest of the study is structured as follows: Section 2 reviews the literature related to this study and provides theoretical hypotheses; Section 3 details data sources, variables, and methodology; Section 4 presents empirical results; Section 5 further analyzes the mediation effects of migrant workers' gender-earning gap between digital finance and their urban integration and the heterogeneity of the effects of digital finance on migrant workers' urban integration; Section 6 presents discussions; Section 7 delves into the conclusions and implications. A technical roadmap is shown in Figure 1.

## 2. Literature background

The New Migration Economics of Labor Migration (NELM) believes that migration decision making is based on the consideration of maximizing household income and minimizing household risks. As a growing number of migrant workers migrate with other family members, they need to enrich their income sources through flexible employment and investment, realize wealth appreciation through investment, and manage their finances so as to accelerate the accumulation of wealth and social resources and afford the increasing living costs in cities. This demand for capital accumulation leads to higher financial demand. Research and recent experience show that digital finance encourages the movement of labor from the agricultural to the non-agricultural sectors by promoting entrepreneurship, investment, and employment, which helps improve migrant workers' operating income and wage income (22). Zhang et al. (18) proposed that inclusive digital finance significantly increases the household income of rural low-income groups and promotes inclusive economic growth in China by promoting the entrepreneurial behavior of rural populations.

Qi and Li (23) further found that the service level of China's digital financial development for young people and women is better than that of other demographic groups. Guo et al. (24) introduced the perspective of gender differences and concluded that FinTech reduces capital constraints and operating costs, thereby promoting women's entrepreneurship and driving more women into employment, reducing the gender wage gap. Zou and Deng (25) examined the relationship between the digital economy and the urban integration of migrants with Chinese cases and found that the digital economy promotes migrants' personal and household income to improve their economic integration and is more conducive to the economic integration of women and new generation migrants. According to the National Bureau of Statistics, since 2014, the proportion of women migrant workers has shown an overall upward trend. In 2021, women migrant workers accounted for 35.9% of all migrant workers. The importance of the urban integration of women migrant workers to the urban integration of all migrant workers rises as their number rises. Gender differences in income levels can be used as a perspective for studying migrant workers' urban integration.

Digital finance gradually reduces female migrant workers' gender vulnerability in the labor market and improves their employability and entrepreneurship (26–28). Guo et al. (24) found that, for each standard deviation increase in digital finance, the growth rate in women's income is 3.42–4.88% greater than that of men, which can hedge the risk of wage decline caused by the increase in women's reproductive and care responsibilities after the birth policy is liberalized. Digital finance provides female migrant workers with a fair opportunity to access financial services. There are more barriers for women to access and use financial services than their male counterparts. Women are considered to be disadvantaged in financial capital due to inherent gender bias and rigid loan policies (29). Digital finance breaks time and space constraints between financial resource demanders and financial resource suppliers. An analysis of historical behavior data of financial service demanders forms credit scores for financing demanders and lowers the financing threshold of the credit market. Research showed that, for every RMB ¥10,000 increase in the per capita assets of rural entrepreneurial households, their entrepreneurial income will increase by RMB ¥1,306 (30). Female migrant workers can obtain credit funds more cheaply and conveniently through online financial services. To some extent, this can ease their financing constraints and support their innovative and entrepreneurial activities.

Additionally, we find that the digital financial demand performance differed by region from the existing research (31). Large cities have unparalleled advantages in financial resources compared to small- and medium-sized cities. With advanced technology, digital finance is rapidly penetrating the geographical space, accelerating the flow of financial elements and forming a wider coverage of inclusive finance, which provides more abundant financial resources for small- and medium-sized cities across time, space, and regions. Therefore, the impact of digital finance on the integration of migrant workers in different cities needs to be considered.

In summary, digital finance can provide female migrant workers not only with equal access to financial services but also with diversified employment opportunities, which enhances their ability to find jobs and start businesses, significantly raising their income level. Therefore, digital finance can significantly increase family income, fill the gender wage gap, and promote family migration. In view of this, the following hypotheses are proposed.

***Hypothesis H1 (H1)***. *Digital finance promotes migrant workers' urban integration*.
***Hypothesis H2 (H2)***. *Digital finance affects migrant workers' urban integration by narrowing the gender gap in their income*.
***Hypothesis H3 (H3)***. *The impact of digital finance on migrant workers' urban integration is more significant for migrant workers located in small and medium-sized cities with low education levels and heavy family burdens*.

## 3. Data, variables, and methodology

### 3.1. Data

The data were from three sources: the digital inclusive finance index, the Chinese Social Survey (CSS), and socioeconomic data from the China Urban Statistical Yearbook.

We used the digital inclusive finance index developed by Peking University to represent the degree of digital finance, which was compiled by the research team of the Digital Finance Research Center of Peking University and the Ant Group Research Institute using Ant Group's massive data on inclusive digital finance. The digital inclusive financial index was obtained from the weighted sum of coverage breadth (54%), usage depth (29.7%), and digitization degree (16.3%) of digital finance. Coverage of digital finance was obtained by the weighted sum of the number of Alipay accounts owned by every 10,000 people, the proportion of Alipay-bound bank card users, and the average number of bank cards bound to each Alipay account. The usage of digital finance was measured by the number of transactions per capita and the number of transactions per capita of digital financial services, such as payment services, monetary fund services, credit services, insurance services, investment services, and credit services, and the number of users using these services per 10,000 Alipay users; the digitalization degree measures the mobility, materialization, credit, and facilitation of digital finance. The higher the proportion of mobile payments in the total number of payments, the lower the interest rate of consumer loans and micro-enterprise loans, and the higher the proportion of deposit-free payments in the total number of payments, the better the value of inclusive digital finance is reflected.

The survey data from CSS in 2019 was used to measure migrant workers' urban integration. The Chinese Social Survey (CSS) is a nationwide biennial survey initiated by the Institute of Sociology of the Chinese Academy of Social Sciences. The survey covered 31 provinces across the country, including 151 districts and counties and 604 villages/neighborhood committees. Each part of the survey included 7,000–10,000 families. In the implementation and management link, the investigation team relied on universities and research institutions all over the country to establish local survey teams, conducted 3–5 days of training courses for supervisors and interviewers and various interview simulation training, designed a series of processes for investigation management, and provided efficient logistics support.

We also collected socioeconomic data from the China Urban Statistical Yearbook so as to better analyze how urban development influences migrant workers' urban integration.

We selected the samples of those who engaged in non-agricultural sectors in urban with agricultural household registration, aged between 16 and 65 years. Then, we constructed the data set for this study by connecting the digital financial index and socioeconomic data with cities where migrant workers lived. The total sample size was 909.

### 3.2. Variables

#### 3.2.1. Dependent variable

Scholars mostly define migrant workers' urban integration based on economical, social, behavioral, psychological, and identity aspects. Considering migration's impact on urban integration in a family way, we proposed a basic framework for the current status of migrant workers' urban integration in terms of economic adaptation, social participation, rights protection, and psychological identity. First, only with a relatively stable job and a fixed residence can migrant workers survive in cities. Economic adaptation is the basis for their full integration into inflow areas. Second, after completing initial survival adaptation, social participation and rights protection are required for migrant workers' urban integration, which reflect their involvement in community activities and social security. Third, profound changes in internal spiritual elements such as new ideas, mentality, and willingness are inevitable results of their adaptation to urban life for a long period of time, reflecting the depth of migrant workers' integration into urban society.

In an exploratory factor analysis of each dimension, Kaiser Meyer Olkin (KMO) was used to compare simple correlation coefficients and partial correlation coefficients among variables, which change between 0 and 1. The closer the KMO value is to 1, the stronger the correlation between variables, and the more suitable the original variables are for factor analysis. However, the KMO values did not exceed 0.6, indicating that factor analysis was not an ideal dimensionality reduction method. Therefore, we estimated the average after summing up each indicator value in Table 1. The higher the score, the higher the degree of migrant workers' urban integration. Their urban integration index system is shown in Table 1.

**Table 1:** Migrant workers' urban integration index system.

**Dimension**	**Indicator**	**Survey items**	**Value quantification**
Economic adaptation	Job stability	How likely is it that you will lose your job in the next 6 months?	Totally possible = 1; Possible = 2; General = 3; Unlikely = 4; Totally impossible = 5
	Housing condition	What type of house do you currently live in?	Dormitory = 1; Private house = 2; Relatives or friends' house = 3; Low-rent house = 4; Self-purchased house = 5
	Job satisfaction	Overall job satisfaction rating	Value 1 corresponds to “very dissatisfied” and 10 corresponds to “very satisfied”
Social participation	Interpersonal relationship	Which of the following online social groups have you joined in the past 2 years?	Only social circles among relatives or none of the above = 1; Social circles among friends, neighbors, countrymen, or colleagues = 2; Interest group, social welfare group, industry group, peer group, association group, or rights protection group = 3
	Volunteering	Have you participated in voluntary activities in the past year?	No = 0; Yes = 1
Rights protection	Employment equity	What do you think of current employment equity?	Very unfair = 1; not fair = 2; fair = 3; very fair = 4
	Wealth and income distribution	What do you think of the current fairness of wealth and income distribution?	Very unfair = 1; not fair = 2; fair = 3; very fair = 4
	Urban insurance	Social security enjoyed by migrant workers	Value 0 corresponds to “none participated” and 5 corresponds to “All five insurances participated”
Psychological identity	Life satisfaction	Overall life satisfaction rating	Value 1 corresponds to “very dissatisfied” and 10 corresponds to “very satisfied”
	Social discrimination	Do you think hukou inequality is serious?	Very serious = 1; relatively serious = 2; serious = 3; not serious = 4
	Subjunctive class identity	Individual subjective class identity change	Changes in individual socioeconomic status compared with 5 years ago

Five insurances refer to pension fund, medical insurance, industrial injury insurance, unemployment insurance, and maternity insurance; Mobile phones and computers have become the main channel for people in China to obtain information, spread information, and engage in other daily behaviors. The indicator “online social groups” can reflect an interpersonal relationship to a certain extent.

#### 3.2.2. Core explanatory variables

The core explanatory variables include individual annual income, the gender-earning gap, and the digital financial index and its coverage breath, usage depth, and digitization degree. Research showed that the income of female migrant workers is lower than that of male migrant workers (20). We subtracted the average annual income of women from the average annual income of men to get the gender-earning gap. The digital inclusive finance index developed by Peking University to reflect the development of digital finance is used in this study (32). The index can be used at three levels: province, municipality, and county. We mainly used the data at the municipal level for regression analysis. To further study the impact of various dimensions of digital finance on migrant workers' urban integration, we also adopted three sub-dimension indicators of digital finance: coverage breadth, usage depth, and digitization degree.

#### 3.2.3. Control variables

For this research, control variables were mainly selected from three aspects: individual, family, and external environment.

We selected age, migration distance, and occupational type variables to reflect personal characteristics. Younger migrant workers have more competitive advantages in job markets, and their employment is stable and formal, which is beneficial to their integration into cities; the economic cost and re-socialization cost of migration increase with an increase in migration distance, which makes it more difficult for migrant workers to integrate into cities (33–35); the informal employment of migrant workers undermines their positions, making it harder for them to enjoy employment rights and social security, which ultimately inhibits their urban integration.

Marital status, the number of family members, and the number of children in the household were chosen as variables to reflect family characteristics. Improving the quality of life of migrant workers' families was our original intention. Married migrant workers with children are more eager to move into cities. Due to the high cost of urban education and the limitation of registered residence status, it is difficult for children with registered residence in rural areas to go to school in cities. Most of them study in their hometowns, which is not conducive to the cultivation of family emotions and children's education. Therefore, married migrant workers with children hope to integrate into cities as soon as possible to achieve family reunification and provide children with better development space.

We chose employment structure, employment capacity, and public service expenditure variables to reflect the characteristics of the external environment. Employment is the primary factor in attracting migrant workers into cities. The size and function of different cities lead to different employment markets. The tertiary industry has greater flexibility and is capable of supplying more jobs. Its weight in GDP represents the employment structure of a city; we used the proportion of the urban working population in the total population to reflect cities' ability to absorb the labor force; local public service can be characterized by the city's expenditure on general public services. Its strengthening is conducive to improving migrant workers' living conditions and promoting their effective integration into cities.

#### 3.2.4. Basic information about the sample

Table 2 shows the descriptive statistics of the sample. Due to regional differences and different economic levels, the development level of digital finance varies greatly, ranging from 206.68 to 308.64. The standard deviation of urban integration was 0.522, which suggested that there was little difference in the urban integration level of migrant workers. The standard deviation of income is far >1, indicating that the income gap for migrant workers is large, ranging from USD $0.435 to USD $290,000. Most of them are middle-aged, with an average age of 40. The education variable's average value was 2.48, and the standard deviation was close to 1, confirming that most migrant workers have a low educational level. Limited by their low educational level, most migrant workers are engaged in low-end manual labor. The occupational type's standard deviation was <1, and the mean value was 4, confirming that most migrant workers are manufacturing and transportation operators. The average values of marital status and the number of children in the household reflect that most migrant workers are married and have children.

**Table 2:** Variable definitions and descriptive statistics.

**Variable**	**Measurement method**	**Mean**	**Std. dev**.	**Min**.	**Max**.
Urban integration	Mean value of all indicators	3.413	0.522	1.833	4.833
Income	Respondent's individual income ($) last year	7,661.048	13,754.86	0.435	290,000
The gender-earning gap	A value obtained by subtracting the average annual income of women from the average annual income of men	1.509	3.250	−2.996	11.513
Digital finance	Digital finance index of the respondent's city	251.803	22.287	206.68	308.64
Age	Age of respondents	40.545	11.886	18	69
Education	Not attending school = 0; Primary school = 1; Junior high school = 2; High school = 3; Junior college = 4; University = 5; Postgraduate = 6	2.48	1.17	0	6
Migration distance	Inter-provincial migration = 1; Inter-city migration = 2; Inter-county migration = 3; Local migrant workers = 4	3.230	1.052	1	4
Occupation type	Managers = 1; Professionals = 2; Commercial-service staff = 3; Operator for Manufacture and Transportation = 4; No fixed position = 5; Others = 6	4.008	0.087	4	5
Marital status	Others = 0; Married = 1	0.794	0.404	0	1
Number of family members	Number of respondents' family members	4.574	1.836	1	14
Number of children in the household	Number of respondents' children	1.407	0.939	0	5
Employment structure	The proportion of tertiary industry in the GDP	0.478	0.094	0.283	0.831
Employment capacity	the proportion of the urban working population in the total population	0.137	0.104	0.037	0.640
Public service expenditure	Urban social security expenditure (USD 1,000,000)	1,828.695	3,149.34	154.058	13,534.15

Income, digital finance, and public service expenditure take logarithms when doing empirical analysis; 2019 annual average exchange rate: 1 yuan = 0.145 US dollars.

### 3.3. Methodology

Based on a theoretical analysis of the impact of digital finance on migrant workers' urban integration, the ordinary least squares (OLS) model is as follows:


(1) $$\begin{array}{l} \text{U}{\text{I}}_{\text{i},\text{c}}\;=\;\alpha_{0}+\alpha_{1}\text{D}{\text{F}}_{\text{c}}+\alpha_{2}\text{contro}{\text{l}}_{\text{i},\text{c}}+\mu_{\text{i},\text{c}} \end{array}$$

where *UI*_*i, c*_ represents urban integration of migrant worker *i* in city *c*; *DF*_*c*_ represents the digital finance development index of the city c;*control*_*i, c*_ represents a series of control variables; μ_*i, c*_ is a random disturbing term; and α_1_ is the corresponding regression coefficient, representing the marginal effect of digital finance development on migrant workers' urban integration. We adopted the ordinary least squares estimation method, which ensured that the model best fits the observed values of the samples and had good predictability.

To test the robustness of the benchmark model, we selected the tool variable method, the bilateral tail reduction method, and the quantile regression method. The tool variable and two-stage least squares method (2SLS) were used to solve the endogeneity problem that may exist in the model. We used the spherical distance from the city where migrant workers live to Hangzhou as the tool variable for digital finance (36). Although the main form of realization of digital finance is online, its degree of development is still affected by geographical and spatial factors. The farther away from Hangzhou, the birthplace of digital economy, the more difficult it is to promote digital finance (37). Distance is related to the development level of digital finance and does not directly affect the urban integration of migrant workers, which conforms to strict exogenous conditions. In addition, because there might be abnormal values in the sample, we conducted bilateral tail reduction at a 1% level for the dependent variable. That is, we found the quantile corresponding to 1% and 99% of each variable, replaced the number less than 1% in the data with 1%, and replaced the number greater than 99% with 99% to smooth the data (the caliber is 1%). Then, we split the data into multiple quantiles according to the dependent variable to study the impact of digital finance on urban integration at different quantiles. We selected five representative quantile points (i.e., the 10th, 30th, 50th, 70th, and 90th quantiles) for quantile regression estimation.

We continued to use the intermediary effect model to test the impact mechanism of digital finance on migrant workers' urban integration. In addition to the direct effect embodied in Equation (1), to discuss the possible mechanisms of digital finance on migrant workers' integration, another verification was conducted to determine whether migrant workers' income and the gender-earning gap are mediating variables between the two. The specific test steps are as follows: (38).


(2) $$\begin{array}{lll} {\text{M}}_{\text{i},\text{c}}\; & = & \;\theta_{0}+\theta_{1}\text{D}{\text{F}}_{\text{c}}+\theta_{2}\text{contro}{\text{l}}_{\text{i},\text{c}}+\mu_{\text{i},\text{c}} \end{array}$$

(3) $$\begin{array}{lll} \text{U}{\text{I}}_{\text{i},\text{c}}\; & = & \;\gamma_{0}+\gamma_{1}\text{D}{\text{F}}_{\text{c}}+\gamma_{2}{\text{M}}_{\text{i},\text{c}}+{\gamma_{3}\text{control}}_{\text{i},\text{c}}+\mu_{\text{i},\text{c}} \end{array}$$

The meaning of the above variables is consistent with equation (1): *M*_*i, c*_ is a mediation variable. We successively selected migrant workers' income and the gender-earning gap as mediation variables to test. α_1_, γ_1_, and θ_1_γ_2_ are the total effect, direct effect, and mediation effect of digital finance on migrant workers' urban integration, respectively.

When α_1_, θ_1_, γ_2_, and γ_1_ are significant, the gender-earning gap plays a partial mediating effect; When α_1_, θ_1_, and γ_2_ are significant, but γ_1_ is not significant, the gender-earning gap plays the complete mediating effect; when α_1_ is significant, and at least one of θ_1_ and γ_2_ is not significant, it is needed to do the Sobel test. If the test is passed, the gender-earning gap will play a mediating role in the process of digital finance affecting migrant workers' urban integration. At a significance level of 0.05, the critical value of the Sobel test is about 0.97.

Finally, we used the grouping regression method to test the regional differences and individual differences in the impact of digital finance on migrant workers' urban integration. The specific groupings are as follows: In terms of regional differences, we divided 284 prefecture-level cities into the south and the north by taking the Qinling Mountains—Huaihai River as the boundary. Furthermore, according to the data of the National Bureau of Statistics of China, 284 prefecture-level cities were divided into two categories: top 100 cities and non-top 100 cities. In terms of individual differences based on education level, we assigned migrant workers without education, with primary education, and with junior high school education to the low education group and migrant workers with high school or above to the high education group. Furthermore, considering that raising children is the main expenditure of families, we classified the sample into two categories according to the number of children in the family: one category was that there is at most one child in the family, and the other category was that there are at least two children in the family.

## 4. Empirical results

### 4.1. Analysis of basic estimation results

We first regressed digital finance and its dimensions on migrant workers' urban integration, and its results are shown in columns (1), (3), (5), and (7) of Table 3. On this basis, we added variables representing characteristics of individuals, families, and the external environment for regression, and its results are shown in columns (2), (4), (6), and (8) of Table 3. After adding control variables, the value and significance of the coefficients of digital finance, usage depth, and digitization degree increased to a certain extent, meaning that we selected reasonable control variables. As shown in columns (1) and (2) of Table 3, digital finance had a positive driving effect on migrant workers' urban integration, with coefficients of 0.599. Further, improvements in usage depth and digitization degree all have significantly positive effects on migrant workers' urban integration, with coefficients of 0.690 and 1.282, respectively. Among them, digitization degree has the highest promotion effect, and coverage breadth is the lowest. To conclude, digital finance significantly promotes migrant workers' urban integration, which verifies H1. For the control variables, the coefficients of migration distance, age, occupation type, and marital status are consistent with our expectations; they indicate that migrant workers with a short migration distance, a younger age, and married status have a higher degree of urban integration but engaging in low-level, repetitive work hinders their integration into cities. The coefficient of the number of children in a household is significantly negative, which indicates that, for migrant workers' families, the burden of raising children is now relatively heavy, and they do not possess enough economic capacity to provide a better environment for children's growth.

**Table 3:** Estimates of the impact of digital finance and its dimensions on migrant workers' urban integration.

	**(1)**	**(2)**	**(3)**	**(4)**	**(5)**	**(6)**	**(7)**	**(8)**
**Dependent variable: Urban integration**								
Digital finance	0.364^*^	0.599^**^						
	(0.199)	(0.304)						
Coverage breadth			0.197	0.179				
			(0.165)	(0.249)				
Usage depth					0.418^**^	0.690^***^		
					(0.172)	(0.226)		
Digitization degree							0.902^***^	1.282^***^
							(0.349)	(0.458)
Age		−0.006^***^		−0.006^***^		−0.007^***^		−0.006^***^
		(0.002)		(0.002)		(0.002)		(0.002)
Migration distance		0.093^***^		0.091^***^		0.095^***^		0.092^***^
		(0.017)		(0.017)		(0.017)		(0.017)
Occupation type		−0.362^*^		−0.356^*^		−0.364^*^		−0.368^*^
		(0.194)		(0.195)		(0.194)		(0.194)
Marital status		0.108^**^		0.112^**^		0.106^**^		0.114^**^
		(0.053)		(0.053)		(0.052)		(0.052)
Number of family members		−0.016		−0.015		−0.017^*^		−0.016
		(0.010)		(0.010)		(0.010)		(0.010)
Number of children in the household		−0.054^**^		−0.057^**^		−0.054^**^		−0.056^**^
		(0.026)		(0.026)		(0.025)		(0.025)
Employment structure		−0.054		−0.024		−0.004		0.051
		(0.222)		(0.226)		(0.219)		(0.219)
Employment capacity		−0.019		0.196		−0.084		−0.003
		(0.260)		(0.257)		(0.237)		(0.228)
Public service expenditure		0.001		0.004		−0.003		−0.017
		(0.019)		(0.019)		(0.019)		(0.021)
*N*	909	909	909	909	909	909	909	909
*R^2^*	0.004	0.068	0.002	0.065	0.006	0.074	0.007	0.072

*, **, *** represent 10, 5, 1% significance levels, respectively; Std. Err. Values are in parentheses.

### 4.2. Robustness tests

#### 4.2.1. Endogenous test

We applied the two-stage least squares (2SLS) method to re-estimate the model, and specific regression results are shown in Table 4. In the first stage of regression, the coefficient of the main variable is significant at the level of 1%, meeting the correlation requirements of the instrumental variable. Moreover, the F-statistic of joint significance is >10, thus passing the test for weak identification. In the second stage of regression, the effect of the development of digital finance on migrant workers' urban integration is still obvious. We used the limited information maximum likelihood (LIML) method for further verification. Its result showed that digital finance significantly drives migrant workers' urban integration, which is in line with the aforementioned consequence. We confirmed that the main conclusion is robust.

**Table 4:** The regression results of the endogenous test.

	**2SLS**		**LIML**
	**First-stage**	**Second-stage**	
Digital finance		2.190^***^	2.190^***^
		(0.534)	(0.535)
The spherical distance between each city and Hangzhou	−0.000^***^		
	(3.62e-06)		
Age	0.000^**^	−0.007^***^	−0.007^***^
	(0.000)	(0.002)	(0.002)
Migration distance	−0.005^***^	0.100^***^	0.100^***^
	(−0.002)	(0.016)	(0.017)
Occupation type	0.018	−0.383^**^	−0.383^*^
	(0.014)	(0.150)	(0.196)
Marital status	0.006	0.094^*^	0.094^*^
	(0.005)	(0.052)	(0.053)
Number of family members	0.003^***^	−0.020^**^	−0.020^*^
	(0.001)	(0.010)	(0.010)
Number of children in the household	−0.007^***^	−0.041	−0.041
	(0.003)	(0.027)	(0.026)
Employment structure	0.178^***^	−0.232	−0.232
	(0.021)	(0.232)	(0.229)
Employment capacity	0.476^***^	−0.900^***^	−0.900^**^
	(0.021)	(0.345)	(0.358)
Public service expenditure	0.001	−0.003	−0.003
	(0.002)	(0.020)	(0.020)
*N*	909	909	909
*R^2^*	0.727	0.040	0.040

*, **, *** represent 10, 5, 1% significance levels, respectively; Std. Err. Values are in parentheses.

#### 4.2.2. Winsorization

We performed the bilateral winsorization of migrant workers' urban integration at a 1% level. As shown in Table 5, the coefficient signs of the core explanatory variables were unchanged and passed the significance test. The impact of digital finance on migrant workers' urban integration is still highly consistent with the basic regression.

**Table 5:** The regression results after winsorization.

	**(1)**	**(2)**	**(3)**	**(4)**
**Dependent variable: Urban integration**				
Digital finance	0.369^*^	0.610^***^	0.561^***^	0.601^**^
	(0.197)	(0.197)	(0.198)	(0.301)
Age		−0.007^***^	−0.006^***^	−0.006^***^
		(0.001)	(0.002)	(0.002)
Migration distance		0.089^***^	0.093^***^	0.093^***^
		(0.016)	(0.016)	(0.017)
Occupation type		−0.423^**^	−0.360^*^	−0.362^*^
		(0.191)	(0.192)	(0.192)
Marital status			0.112^**^	0.112^**^
			(0.052)	(0.052)
Number of family members			−0.015	−0.015
			(0.010)	(0.010)
Number of children in the household			−0.056^**^	−0.056^**^
			(0.025)	(0.025)
Employment structure				−0.050
				(0.219)
Employment capacity				−0.019
				(0.258)
Public service expenditure				0.001
				(0.019)
*N*	909	909	909	909
*R^2^*	0.004	0.058	0.070	0.070

*, **, *** represent 10, 5, 1% significance levels, respectively; Std. Err. Values are in parentheses.

#### 4.2.3. Quantile regression analysis

The results of quantile regression are shown in Table 6. Digital finance positively affects migrant workers' urban integration at different quantile points, and the effect is statistically significant at the 30th and 50th quantiles. The research conclusion is still robust.

**Table 6:** The results of quantile regression.

	**10th**	**30th**	**50th**	**70th**	**90th**
Digital finance	0.390	1.322^***^	0.856^**^	0.354	0.466
	(0.457)	(0.439)	(0.393)	(0.428)	(0.496)
Age	−0.008^**^	−0.008^**^	−0.007^***^	−0.007^**^	−0.003
	(0.003)	(0.003)	(0.002)	(0.003)	(0.003)
Migration distance	0.075^**^	0.089^***^	0.093^***^	0.123^***^	0.118^***^
	(0.035)	(0.025)	(0.022)	(0.023)	(0.024)
Occupation type	−0.197	−0.334^**^	−0.492^**^	−0.345	−0.309
	(0.172)	(0.151)	(0.244)	(0.268)	(0.294)
Marital status	−0.085	0.151^**^	0.194^***^	0.249^**^	0.137^*^
	(0.071)	(0.075)	(0.064)	(0.104)	(0.073)
Number of family members	−0.024	−0.018	−0.018	−0.027	−0.007
	(0.016)	(0.013)	(0.013)	(0.019)	(0.014)
Number of children in the household	−0.024	−0.055	−0.066^**^	−0.091^**^	−0.100^***^
	(0.047)	(0.038)	(0.027)	(0.042)	(0.038)
Employment structure	−0.130	−0.241	−0.0729	0.155	0.205
	(0.328)	(0.304)	(0.344)	(0.334)	(0.343)
Employment capacity	0.358	−0.338	−0.171	0.108	−0.236
	(0.476)	(0.383)	(0.434)	(0.321)	(0.378)
Public service expenditure	−0.001	0.005	0.011	−0.001	0.010
	(0.032)	(0.030)	(0.025)	(0.033)	(0.034)
*N*	909	909	909	909	909
*R^2^*	0.042	0.042	0.042	0.047	0.046

*, **, *** represent 10, 5, 1% significance levels, respectively; Std. Err. Values are in parentheses.

## 5. Further analysis

### 5.1. Intermediary effect tests

#### 5.1.1. Intermediary effect test of migrant workers' income

Models (2), (6), and (8) of Table 3 were used to examine the total effects of digital finance, its usage depth, and digitization degree on migrant workers' urban integration; models (1), (3), and (5) of Table 7 were adopted to examine the effects of digital finance, its usage depth, and digitization degree on migrant workers' income; model (2), (4), and (6) of Table 7 were used to examine the effects of migrant workers' urban integration on digital finance and their income.

**Table 7:** Results of the effect of digital finance on migrant workers' urban integration (mediation effect of their income).

**Variables**	**(1)**	**(2)**	**(3)**	**(4)**	**(5)**	**(6)**
	**Income**	**Urban** **integration**	**Income**	**Urban** **integration**	**Income**	**Urban** **integration**
Digital finance	1.980^***^	0.401				
	(0.586)	(0.300)				
Usage depth			1.605^***^	0.533^**^		
			(0.436)	(0.223)		
Digitization degree					1.298	1.151^**^
					(0.890)	(0.450)
Migrant workers' income		0.100^***^		0.098^***^		0.101^***^
		(0.017)		(0.017)		(0.017)
Age	−0.018^***^	−0.004^***^	−0.018^***^	−0.005^***^	−0.017^***^	−0.004^***^
	(0.003)	(0.002)	(0.003)	(0.002)	(0.003)	(0.002)
Migration distance	−0.094^***^	0.102^***^	−0.092^***^	0.104^***^	−0.101^***^	0.102^***^
	(0.032)	(0.016)	(0.032)	(0.016)	(0.032)	(0.016)
Occupation type	−0.115	−0.350^*^	−0.111	−0.353^*^	−0.102	−0.357^*^
	(0.375)	(0.191)	(0.374)	(0.190)	(0.377)	(0.190)
Marital status	0.379^***^	0.0704	0.379^***^	0.069	0.397^***^	0.074
	(0.101)	(0.052)	(0.101)	(0.052)	(0.102)	(0.052)
Number of family members	−0.004	−0.015	−0.005	−0.016	−0.001	−0.016
	(0.019)	(0.010)	(0.019)	(0.010)	(0.020)	(0.010)
Number of children in the household	−0.132^***^	−0.041	−0.137^***^	−0.040	−0.146^***^	−0.042^*^
	(0.049)	(0.025)	(0.049)	(0.025)	(0.049)	(0.025)
Employment structure	−0.664	0.012	−0.481	0.043	−0.403	0.092
	(0.428)	(0.218)	(0.422)	(0.215)	(0.426)	(0.215)
Employment capacity	−0.062	−0.013	0.113	−0.095	0.715	−0.075
	(0.502)	(0.256)	(0.457)	(0.232)	(0.443)	(0.224)
Public service expenditure	0.007	0.001	−0.001	−0.003	−0.008	−0.016
	(0.037)	(0.019)	(0.037)	(0.019)	(0.040)	(0.020)
*N*	909	909	909	909	909	909
*R^2^*	0.109	0.103	0.111	0.107	0.099	0.108

*, **, *** represent 10, 5, 1% significance levels, respectively; Std. Err. Values are in parentheses.

It was observed that the coefficients of digital finance, its usage depth, and digitization degree on migrant workers' urban integration are positive. The coefficients of digital finance and its usage depth on migrant workers' income are observed to be positive. As is shown in models (2) and (4), after controlling digital finance, migrant workers' income is found to have positive effects on their urban integration, indicating that migrant workers' income has mediation effects. It was determined that digital finance and its usage depth have direct positive effects (0.401 and 0.533) and mediation effects (0.198 and 0.157) on migrant workers' urban integration separately. Further, the mediation effects accounted for 33.06 and 22.8% of the total effects, respectively. The degree of digitization has no significant effect on the migrant workers' income. Therefore, tests to determine whether or not mediation effects exist were continued. In accordance with the results of the Sobel test, the statistical value of *Z* was determined to be 1.42, which is greater than the critical value of 0.97 at the significance level of 5%. Therefore, it is concluded that digital finance and its usage depth and digitization degree affected migrant workers' urban integration through their income.

#### 5.1.2. Intermediary effect test of the gender-earning gap

From the results in Table 8, we can see that there is a significant gender difference in the correlation between the development of digital finance and the income level of migrant workers. Digital finance and its usage depth and digitization degree significantly improved migrant workers' income, and it was more significant for female migrant workers. When one unit of digital finance is added, the income of female migrant workers increases by 303.1%, making it higher than that of male migrant workers. When one unit of usage depth is added, the income of female migrant workers increases by 319.4%. Digitization degree only significantly improves migrant women's income. For female migrant workers, the development and popularization of digital finance provide financial support for their entrepreneurship, help stimulate entrepreneurial vitality, and improve labor participation and income levels. In contrast, due to wider financing channels, the change in male migrant workers' income is less affected by digital finance, thus narrowing the gender-earning gap, which preliminarily validates H2.

**Table 8:** Results of the effect of digital finance on migrant workers' urban integration (mediation effect of their income).

**Variables**	**(1)** **Female**	**(2)** **Male**	**(3)** **Female**	**(4)** **Male**	**(5)** **Female**	**(6)** **Male**
**Dependent variable: Income**						
Digital finance	3.031^***^	1.587^**^				
	(0.994)	(0.670)				
Usage depth			3.194^***^	1.128^**^		
			(0.772)	(0.484)		
Digitization degree					3.269^**^	1.188
					(1.513)	(1.020)
Age	−0.023^***^	−0.023^***^	−0.025^***^	−0.023^***^	−0.022^***^	−0.023^***^
	(0.006)	(0.004)	(0.006)	(0.004)	(0.006)	(0.004)
Migration_istance	−0.152^***^	−0.041	−0.138^**^	−0.041	−0.165^***^	−0.044
	(0.056)	(0.036)	(0.056)	(0.036)	(0.056)	(0.036)
Occupation	0.128	−0.067	0.167	−0.063	0.065	−0.022
	(0.609)	(0.441)	(0.602)	(0.441)	(0.612)	(0.442)
Married	0.257	0.617^***^	0.244	0.626^***^	0.266	0.643^***^
	(0.167)	(0.122)	(0.165)	(0.122)	(0.168)	(0.122)
Family_size	−0.011	−0.02	−0.0167	−0.02	−0.005	−0.019
	(0.032)	(0.023)	(0.032)	(0.023)	(0.032)	(0.023)
Kids	−0.121	−0.108^*^	−0.122	−0.115^*^	−0.134^*^	−0.122^**^
	(0.080)	(0.059)	(0.079)	(0.059)	(0.08)	(0.059)
Employment structure	0.172	−1.147^**^	0.598	−1.027^**^	0.704	−0.946^*^
	(0.713)	(0.491)	(0.702)	(0.484)	(0.723)	(0.485)
Employment capacity	−0.867	0.410	−1.030	0.640	−0.026	1.008^**^
	(0.855)	(0.572)	(0.785)	(0.517)	(0.764)	(0.500)
Public service expenditure	0.061	−0.034	0.036	−0.036	0.009	−0.045
	(0.069)	(0.041)	(0.069)	(0.041)	(0.073)	(0.044)
Observations	370	539	370	539	370	539
R-squared	0.156	0.147	0.173	0.147	0.145	0.140

*, **, *** represent 10, 5, 1% significance levels, respectively; Std. Err. Values are in parentheses.

Models (2), (6), and (8) of Table 3 were used to examine the total effects of digital finance and its usage depth and digitization degree on migrant workers' urban integration; Models (1), (3), and (5) of Table 9 were adopted to examine the effects of digital finance and its usage depth and digitization degree on migrant workers' gender-earning gaps; models (2), (4), and (6) of Table 9 were used to examine the effects of migrant workers' urban integration on digital finance and the gender-earning gap.

**Table 9:** Results of the effect of digital finance on migrant workers' urban integration (mediation effect of their gender-earning gap).

	**(1)** **Gender-earning**	**(2)** **Urban**	**(3)** **Gender-earning**	**(4)** **Urban**	**(5)** **Gender-earning**	**(6)** **Urban**
Digital finance	−5.967^***^	0.529^*^				
	(1.896)	(0.305)				
Usage depth			−7.489^***^	0.614^***^		
			(1.398)	(0.229)		
Digitization degree					−10.640^***^	1.165^**^
					(2.859)	(0.461)
gender-earning gap		−0.012^**^		−0.010^*^		−0.011^**^
		(0.005)		(0.005)		(0.005)
Age	0.018^*^	−0.006^***^	0.022^**^	−0.006^***^	0.017	−0.006^***^
	(0.011)	(0.002)	(0.011)	(0.002)	(0.011)	(0.002)
Migration distance	−0.224^**^	0.090^***^	−0.249^**^	0.092^***^	−0.215^**^	0.090^***^
	(0.104)	(0.017)	(0.103)	(0.017)	(0.104)	(0.017)
Occupation type	−1.606	−0.381^*^	−1.576	−0.380^*^	−1.572	−0.385^**^
	(1.212)	(0.194)	(1.200)	(0.194)	(1.210)	(0.194)
Marital status	0.005	0.108^**^	0.0329	0.107^**^	−0.0508	0.113^**^
	(0.328)	(0.052)	(0.325)	(0.052)	(0.327)	(0.052)
Number of family members	0.103	−0.015	0.115^*^	−0.016	0.103^*^	−0.015
	(0.063)	(0.010)	(0.062)	(0.010)	(0.063)	(0.010)
Number of children in the household	−0.245	−0.057^**^	−0.250	−0.056^**^	−0.216	−0.059^**^
	(0.159)	(0.026)	(0.157)	(0.025)	(0.159)	(0.025)
Employment structure	−3.811^***^	−0.099	−4.297^***^	−0.047	−4.796^***^	−0.001
	(1.384)	(0.222)	(1.354)	(0.219)	(1.367)	(0.220)
Employment capacity	1.287	−0.004	2.284	−0.060	0.598	0.004
	(1.624)	(0.260)	(1.466)	(0.237)	(1.422)	(0.228)
Public service expenditure	−0.308^**^	−0.002	−0.262^**^	−0.005	−0.162	−0.018
	(0.121)	(0.019)	(0.120)	(0.019)	(0.128)	(0.021)
*N*	909	909	909	909	909	909
*R^2^*	0.065	0.073	0.084	0.078	0.069	0.077

*, **, *** represent 10, 5, 1% significance levels, respectively; Std. Err. Values are in parentheses.

It was found that the regression results of models (2), (6), and (8) of Table 3 indicated that, after controlling a series of other variables, digital finance, its usage depth, and digitization degree have significant positive effects on migrant workers' urban integration. These findings suggest that digital finance, its usage depth, and its digitization degree effectively encouraged migrant workers' urban integration. The empirical results of models (1), (3), and (5) of Table 9 demonstrate that digital finance and its usage depth and digitization degree have negative impacts on migrant workers' gender-earning gaps, suggesting that growing financial inclusiveness will narrow gender differences in migrant workers' income. The empirical results of models (2), (4), and (6) of Table 9 indicated that there are negative correlations between migrant workers' gender-earning gap and their urban integration. This confirms that the mediation effect exists. Digital finance and its usage depth and digitization degree have separate direct effects (0.529, 0.614, and 1.165) and mediation effects (0.072, 0.075, and 0.117) on migrant workers' urban integration. Further, the mediation effects accounted for 11.95, 10.85, and 9.13% of the total effects, respectively. Overall, digital finance and its usage depth and digitization degree promote migrant workers' urban integration through the mediation effects of their gender-earning gap, which verifies H2.

### 5.2. Heterogeneity analysis

#### 5.2.1. Heterogeneity analysis of urban resource endowment

To test whether there are regional differences in the impact of digital finance on migrant workers' urban integration, the 284 prefecture-level cities are divided into northern and southern regions for regression estimation. It is observed that, in the test for the southern region, digital finance plays a significant positive role in promoting migrant workers' urban integration at the 1% level. In contrast, in the test for the northern region, the role of digital finance on migrant workers' urban integration is not significant, which does not reflect the inclusiveness of digital finance.

Furthermore, the 284 prefecture-level cities are classified into “top 100 cities” and “non-top 100 cities” for regression estimation. The results are presented in columns (3) and (4) of Table 10, showing that digital finance significantly promotes migrant workers' urban integration working in non-top 100 cities. Nevertheless, this finding does not hold for the top 100 cities. The reason is that the emergence of digital finance and innovative financial models make up for the shortcomings of the traditional financial system in serving non-top 100 cities and contribute significantly to diversifying China's financial market. When the economic environment is significantly improved, migrant workers in small- and medium-sized cities quickly seize the opportunities offered by digital finance and access more external financing to accelerate their urban integration. Thus, the role of digital finance in encouraging migrant workers' urban integration is more clear in non-top 100 cities, which partly verifies H3.

**Table 10:** Regression results of heterogeneity analysis.

	**(1)**	**(2)**	**(3)**	**(4)**
	**Northern region**	**Southern region**	**Top 100 cities**	**Non-top 100 cities**
Digital finance	0.431	1.028^***^	−0.523	0.760^*^
	(0.616)	(0.375)	(0.507)	(0.454)
Age	−0.009^***^	−0.005^**^	−0.005^*^	−0.008^***^
	(0.003)	(0.002)	(0.002)	(0.002)
Migration distance	0.095^***^	0.088^***^	0.113^***^	0.060^**^
	(0.034)	(0.020)	(0.024)	(0.024)
Occupation type	0.253	−0.517^**^	−0.375	−0.378
	(0.511)	(0.211)	(0.295)	(0.258)
Marital status	0.205^**^	0.027	0.096	0.100
	(0.091)	(0.065)	(0.077)	(0.071)
Number of family members	−0.043^**^	−0.002	0.001	−0.021
	(0.021)	(0.012)	(0.016)	(0.013)
Number of children in the household	−0.040	−0.049	−0.069^*^	−0.038
	(0.048)	(0.030)	(0.037)	(0.036)
Employment structure	0.389	−0.367	0.685^*^	−0.120
	(0.423)	(0.267)	(0.405)	(0.273)
Employment capacity	−0.156	−0.074	0.536	−0.404
	(0.541)	(0.302)	(0.352)	(0.504)
Public service expenditure	0.015	−0.002	−0.082^***^	0.050
	(0.044)	(0.022)	(0.029)	(0.047)
*N*	323	586	440	469
*R^2^*	0.073	0.081	0.091	0.066

*, **, *** represent 10, 5, 1% significance levels, respectively; Std. Err. Values are in parentheses.

#### 5.2.2. Heterogeneity analysis of personal characteristics

We compared migrant workers with different education levels and the number of children they have, and we analyzed the individual heterogeneity of the impact of digital finance on migrant workers' urban integration. The empirical results in Table 11 show that migrant worker families with low human capital and two or more children benefit more from the development of digital finance. The incentive effects of digital finance on the urban integration of migrant workers with low education levels and two or more children are significantly positive correlations, with coefficients of 0.988 and 1.147, respectively. In contrast, for migrant workers with high education levels and, at most, one child, the coefficients of digital financial inclusion are not significant. Digital finance reduces the financing threshold, and entrepreneurial costs significantly ease liquidity constraints and stimulate investment and business activities. Their economic capabilities improve significantly. Thus, the role of digital finance in promoting migrant workers' urban integration is more clear for migrant workers with low education levels and two or more children, which finally verifies H3.

**Table 11:** Regression results of heterogeneity analysis.

	**(1)**	**(2)**	**(3)**	**(4)**
	**High educational** **level**	**Low educational** **level**	**Number of** **kids ≤ 1**	**Number of** **kids ≥ 2**
Digital finance	−0.369	0.988^***^	0.173	1.147^**^
	(0.473)	(0.374)	(0.422)	(0.444)
Age	−0.002	−0.003	−0.007^***^	−0.006^***^
	(0.003)	(0.002)	(0.002)	(0.002)
Migration distance	0.069^***^	0.101^***^	0.098^***^	0.09^***^
	(0.025)	(0.021)	(0.023)	(0.025)
Occupation type	0.122	−0.500^**^	−0.112	−0.432^*^
	(0.468)	(0.210)	(0.370)	(0.237)
Marital status	0.248^***^	−0.074	0.079	0.154
	(0.078)	(0.071)	(0.057)	(0.135)
Number of family members	−0.028^*^	−0.010	−0.021	−0.013
	(0.015)	(0.013)	(0.016)	(0.013)
Number of children in the household	−0.047	−0.013	–	–
	(0.045)	(0.030)	–	–
Employment structure	−0.131	−0.058	0.132	−0.322
	(0.328)	(0.284)	(0.333)	(0.303)
Employment capacity	0.527	−0.266	0.270	−0.410
	(0.390)	(0.333)	(0.366)	(0.383)
Public service expenditure	0.015	−0.010	−0.002	−0.004
	(0.028)	(0.025)	(0.027)	(0.029)
*N*	367	542	460	449
*R^2^*	0.068	0.071	0.054	0.067

*, **, *** represent 10, 5, 1% significance levels, respectively; Std. Err. Values are in parentheses.

## 6. Discussions

This study revealed that the development of digital finance could alleviate the inequality of female migrant workers' rights and abilities to create wealth, narrow the gender gap in income, and promote family migration. The empirical results were consistent with those of migrant workers' cases about rural-urban migration (39, 40). However, unlike previous studies (39, 40), our study paid more attention to the impact of digital finance on migrant workers' urban integration because of the gender-earning gap.

(1) *Digital finance mainly affects migrant workers' urban integration by narrowing the gender income gap among migrant workers*

The results of the endogenous test, winsorization, and quantile regression analysis showed that digital finance urban integration, which is in line with previous studies digital finance breaks the constraints of time and space on the employment of migrant workers, enables women to balance work and family, and gives them flexible and free working hours, which helps reduce gender discrimination. Second, digital finance amplifies women's emotional advantages and expands their value. For example, women are more capable of understanding consumer needs and perceiving the market. With the integration of digital finance, these soft-skill advantages become employment advantages for women. Third, female migrant workers can obtain guaranteed loans for entrepreneurship through online financial services, alleviating the financing constraints of female migrant workers and enhancing women's willingness to start businesses. However, it had no significant impact on the entrepreneurial behavior of male migrant workers with wider access to financing and fewer capital constraints. In the case where the income of female migrant workers is lower than that of male migrant workers, digital finance adds 1 unit, and the income of female migrant workers increases by 303.1%, which is higher than that of male migrant workers. The role of digital finance in promoting the income growth of female migrant workers is significantly positive and will bridge the gender income gap of migrant workers, thus confirming the “digital dividend” effect brought by the development of digital finance. effect brought by the development of digital finance.

(2) *The usage depth and digitization degree of digital finance have significant impacts on migrant workers' urban integration but a strong effect on lower-income families with more female members*.

The intermediary effect test of migrant workers' income and the gender-earning gap further prove the coefficients of digital finance, and its usage depth and digitization degree on migrant workers' income are observed to be positive. Prior studies (41, 42) confirmed that low-income farming households have a higher demand for credit and more difficult financing, and there is a greater need for digital financial services to ease financing constraints and indirectly improve income and quality of life while increasing the labor force participation of female migrant workers. Digital finance and its usage depth and digitization degree significantly improve migrant workers' incomes, and this is especially true for female migrant workers. However, research shows that simply emphasizing the coverage of digital finance, such as by simply opening digital accounts, accounts, does not have much effect on increasing women's incomes or urban integration. It is important to provide additional programs on guide female migrant workers to improve their digital operation capabilities and enhance their ability to access financial services through digital financial service platforms. This will help increase their incomes, thereby promoting the urban integration of migrant workers.

(3) *Migrant workers' urban integration depends on the interaction of many factors. A high level of digital finance does not represent a high urban integration capacity of migrant workers, but the high degree of urban integration of migrant workers can reflect the good level of development of the digital financial market in the region*. the region.

There are regional differences in digital finance, especially between the southern cities and the northern cities. A heterogeneity analysis of urban resource endowment showed that there are regional differences in the impact of digital finance on migrant workers' urban integration. Compared with the northern region, in the test in the southern region, digital finance had a more significant positive effect on promoting the integration of migrant workers into the city. Namely, different from traditional finance, digital finance has a weak spillover effect on regional population mobility, economic development, and industrial structure due to geographical distance (43–45). The improvement of the development level of the digital financial market causes blockchain disruption and decentralized finance, eases labor capital constraints, and improves labor employability. Migrant workers actively seize the development opportunities of digital finance, quickly obtain financing, and carry out innovative and entrepreneurial behaviors so as to further improve migrant workers' urban integration.

## 7. Conclusion and implications

This research analyzed the impact of income on migrant workers' urban integration in the context of digital finance and analyzed the role of digital finance in increasing the income of female migrant workers, filling the gender gap in income, and promoting family migration from the perspective of gender differences. The key findings of this study are organized as follows:

(1) Digital finance and its usage depth and digitalization degree significantly improved female migrant workers' income. When 1 unit of digital finance is added, the income of female migrant workers increases by 303.1%, higher than that of male migrant workers; 1 unit of usage depth increases the income of female migrant workers by 2.066% greater than that of male migrant workers; digitalization degree has a significant impact on the income of female migrant workers but not that of male migrant workers; an increase in age decreases the income of both male or female migrant workers.
(2) Digital finance and its usage depth and digitalization degree affect the urban integration of migrant workers by reducing their gender income gap. When usage depth increases by 1 unit, the gender difference in migrant workers' income decreases by 7.489 units, and the level of their urban integration increases by 0.614 units. Increasing the digitization degree by 1 unit results in a 10.64 unit decrease in the gender-earning gap and a 1.165 unit increase in urban integration.

Our results have several policy implications for narrowing migrant workers' gender-earning gap and promoting their urban integration. First, the government should further improve the top-level design and fully release the digital gender dividend, introducing a gender perspective when formulating relevant policies to support digital finance, flexible employment, mass entrepreneurship, and more. Second, it is necessary to make investment to individual or a certain group who needs digital financial products. The government needs to conduct precision marketing through big data analysis, increase digital finance and product services that are more popular with female migrant workers, and continuously launch new products and services for female migrant workers to improve the stickiness and number of such customers. Moreover, the government should expand the construction of digital infrastructure and promote the digital transformation of the banking industry, further reducing the loan threshold for female migrant workers; third, we should accord importance to the training of women's skills in using digital finance, employment, and entrepreneurship and help more women transform from traditional laborers to practitioners of digital economy. At present, there are a large number of female migrant workers in small- and medium-sized cities, county towns, and other sinking markets in China who urgently need training in the use of digital finance. Therefore, we should strengthen women workers' digital skills training and entrepreneurship training, especially to provide more active digital financial services for female migrant workers in low- and medium-skilled jobs.

Studying the impact of digital finance on migrant workers' urban integration is of great significance both in terms of academic value and policy formulation. However, the study also has certain limitations. There are many factors that affect migrant workers' family migration decisions. When we attempted to select control variables, we were limited by the availability of survey data. There may be an incomplete selection of variables, which needs to be further improved in subsequent research. In addition, we used 1-year data and did not discuss in depth the dynamic impact of digital finance on gender differences in migrant workers' income and urban integration. Further, researchers need to reveal the characteristics of the dynamic evolution of the impact of income on migrant workers' urban integration in the context of digital finance.

## Data availability statement

The original contributions presented in the study are included in the article/Supplementary material, further inquiries can be directed to the corresponding author.

## Author contributions

ZY and TL contributed to the conception and design of the work. ZY designed the study and collected the data. ZY, TL, and YX drafted and revised the manuscript, which was then finalized by ZY and YX. All authors commented on drafts of the manuscript and approved the final manuscript.
