# Supplementary material for: Digital finance and migrant workers' urban integration: The mediation effect of the gender-earning gap
Source: Front Public Health. 2022 Dec 19;10:1076783. doi: 10.3389/fpubh.2022.1076783 (PMC9806269; doi:10.3389/fpubh.2022.1076783)
Supplement: Supplementary file 1 [file Table_1.docx]

# Appendix Table

Here we give more detailed information of the sample. The statistical results show that annual income of female migrant workers is lower than that of male migrant workers, and the former only accounts for 84.5% of the latter. Nearly 80% of migrant workers are married and have 1-2 children. Family migration and short-range migration are the main trend of population mobility. Most men and women migrate with their spouses, accounting for 69.02% and 68.11% respectively. In terms of human capital, female migrant workers are younger, with an average age of 39; Migrant workers are mainly junior high school graduates, and the proportion of female migrant workers who not attended school and are primary school graduates is higher than that of men, while the proportion of female migrant workers who are junior high school graduates is lower than that of men; On the whole, the majority of migrant workers didn’t receive any on-the-job training, indicating a lack of human capital accumulation of migrant workers. In terms of employment, migrant workers are mostly engaged in services, manufacturing and other low skill threshold jobs, working for private and individual owners; Private and individual owners do not attach importance to protecting migrant workers’ labor rights and interests. The results show that more than 60% of migrant workers didn’t sign labor contracts with employment units; About 30% of migrant workers consider that employment is unfair, and female migrant workers are 2.3 percentage points higher than men.

Appendix **Table** Basic information of male migrant workers and female migrant workers.

| **Variable** | **ALL** | **Male** | **Female** | **Variable** | **ALL** | **Male** | **Female** |
| --- | --- | --- | --- | --- | --- | --- | --- |
|  | **909** | **539** | **370** |  | **909** | **539** | **370** |
| **Annual income ($)** | | | | **Contract signing (%)** | | | |
| Average value | 7661.05 | 8177.02 | 6909.4 | Signed labor contract | 35.31% | 34.51% | 36.49% |
| **Age (year)** | | | | Others | 2.75% | 2.78% | 2.70% |
| Average value | 40.54 | 41.61 | 39 | No contract signed | 61.94% | 62.71% | 60.81% |
| **Education (%)** | | | | **Number of children in household(%)** | | | |
| Not attending school | 2.20% | 1.67% | 2.97% | 1 | 31.24% | 28.01% | 35.95% |
| Primary school | 14.41% | 13.73% | 15.41% | 2 | 40.48% | 40.63% | 40.27% |
| Junior high school | 43.01% | 46.20% | 38.38% | More than 3 | 8.91% | 8.72% | 9.19% |
| High school | 22.11% | 22.08% | 22.16% | No children | 19.36% | 22.63% | 14.59% |
| Junior college degree or above | 18.26% | 16.33% | 21.08% | **Migration distance (%)** | | | |
| **Marital status (%)** | | | | Inter-provincial migration | 10.78% | 11.87% | 9.19% |
| Unmarried | 20.57% | 23.93% | 15.68% | Inter-city migration | 14.08% | 14.10% | 14.05% |
| Married | 79.43% | 76.07% | 84.32% | Inter-county migration | 16.50% | 13.73% | 20.54% |
| **Fairness of employment (%)** | | | | Local migrant workers | 58.64% | 60.30% | 56.22% |
| Very unfair | 4.40% | 4.45% | 4.32% | **Possibility of unemployment in the next six months (%)** | | | |
| Unfair | 27.28% | 26.35% | 28.65% | Totally possible | 8.80% | 8.35% | 9.46% |
| Fair | 58.20% | 59.18% | 56.76% | Possible | 17.71% | 17.81% | 17.57% |
| Very fair | 10.12% | 10.02% | 10.27% | General | 9.46% | 9.46% | 9.46% |
| **Skill requirements (%)** | | | | Unlikely | 30.03% | 28.01% | 32.97% |
| Very highly skilled | 8.25% | 10.02% | 5.68% | Totally impossible | 33.99% | 36.36% | 30.54% |
| Highly skilled | 14.85% | 18.74% | 9.19% | **Work unit (%)** | | | |
| General skill | 40.81% | 40.63% | 41.08% | Governmental agency | 1.54% | 1.67% | 1.35% |
| No professional skills | 36.08% | 30.61% | 44.05% | State-controlled enterprises | 5.50% | 7.24% | 2.97% |
| **On-the-job training (%)** | | | | Collective institutions | 6.05% | 4.27% | 8.65% |
| No training | 73.27% | 77.92% | 66.49% | Collective enterprise | 2.20% | 2.04% | 2.43% |
| Training available | 26.73% | 22.08% | 33.51% | Private enterprise | 35.42% | 35.62% | 35.14% |
| **Migration mode (%)** | | | | Foreign enterprise | 1.87% | 1.30% | 2.70% |
| Move with own spouse | 68.65% | 69.02% | 68.11% | Individual businesses | 26.62% | 25.05% | 28.92% |
| Move with other family members | 17.82% | 15.21% | 21.62% | Other enterprises | 20.79% | 22.82% | 17.84% |
| Self-migration | 13.53% | 15.77% | 10.27% |  |  |  |  |
